# Author Correction: Concordance of randomised controlled trials for artificial intelligence interventions with the CONSORT-AI reporting guidelines

**DOI:** 10.1038/s41467-024-49956-w

**Published:** 2024-07-29

**Authors:** Alexander P. L. Martindale, Carrie D. Llewellyn, Richard O. de Visser, Benjamin Ng, Victoria Ngai, Aditya U. Kale, Lavinia Ferrante di Ruffano, Robert M. Golub, Gary S. Collins, David Moher, Melissa D. McCradden, Lauren Oakden-Rayner, Samantha Cruz Rivera, Melanie Calvert, Christopher J. Kelly, Cecilia S. Lee, Christopher Yau, An-Wen Chan, Pearse A. Keane, Andrew L. Beam, Alastair K. Denniston, Xiaoxuan Liu

**Affiliations:** 1 Brighton and Sussex Medical School, Brighton, UK; 2 Department of Primary Care and Public Health, Brighton and Sussex Medical School, Brighton, UK; 3 Birmingham and Midland Eye Centre, Sandwell and West Birmingham NHS Trust, Birmingham, UK; 4 Christ Church, University of Oxford, Oxford, UK; 5 University College London Medical School, London, UK; 6 Institute of Inflammation and Ageing, University of Birmingham, Birmingham, UK; 7 University Hospitals Birmingham NHS Foundation Trust, Birmingham, UK; 8 National Institute for Health and Care Research (NIHR) Birmingham Biomedical Research Centre, University of Birmingham, Birmingham, UK; 9 York Health Economics Consortium, University of York, Birmingham, UK; 10 Northwestern University Feinberg School of Medicine, Chicago, Illinois USA; 11 Centre for Statistics in Medicine//UK EQUATOR Centre, Nuffield Department of Orthopaedics, Rheumatology and Musculoskeletal Sciences, University of Oxford, Oxford, UK; 12 Centre for Journalology, Clinical Epidemiology Program, Ottawa Hospital Research Institute, Ottowa, Canada; 13 Department of Bioethics, The Hospital for Sick Children, Toronto, Canada; 14 Genetics & Genome Biology Research Program, Peter Gilgan Centre for Research & Learning, Toronto, Canada; 15 Division of Clinical and Public Health, Dalla Lana School of Public Health, Toronto, Canada; 16 Australian Institute for Machine Learning, University of Adelaide, Adelaide, Australia; 17 Birmingham Health Partners Centre for Regulatory Science and Innovation, University of Birmingham, Birmingham, UK; 18 Centre for Patient Reported Outcomes Research (CPROR), Institute of Applied Health Research, College of Medical and Dental Sciences, University of Birmingham, Birmingham, UK; 19 NIHR Applied Research Collaboration (ARC) West Midlands, University of Birmingham, Birmingham, UK; 20 NIHR Blood and Transplant Research Unit (BTRU) in Precision Transplant and Cellular Therapeutics, University of Birmingham, Birmingham, UK; 21 Google Health, London, UK; 22 University of Washington, Seattle, WA USA; 23 Nuffield Department of Women’s and Reproductive Health, University of Oxford, Oxford, UK; 24 Health Data Research UK, London, UK; 25 Department of Medicine, Women’s College Hospital. University of Toronto, Toronto, Canada; 26 NIHR Biomedical Research Centre at Moorfields, Moorfields Eye Hospital NHS Foundation Trust and UCL Institute of Ophthalmology, London, UK; 27 Department of Epidemiology, Harvard. T.H. Chan School of Public Health, Boston, MA USA; 28 Department of Biomedical Informatics, Harvard Medical School, Boston, MA USA

**Keywords:** Clinical trial design, Technology, Health policy

Correction to: *Nature Communications* 10.1038/s41467-024-45355-3, published online 22 February 2024

The original version of this Article omitted from the author list the 2nd and 3rd authors, Carrie D. Llewellyn and Richard O. de Visser, respectively. Carrie D. Llewellyn and Richard O. de Visser are from the Department of Primary Care and Public Health, Brighton and Sussex Medical School, UK. Consequently, the following was added to the Author Contributions: ‘CDL, ROdV: supervision, writing (original draft, review & editing)’; and ‘LFR, RG, GSC, DM, MM, LOR, SCR, MC, CK, CDL, CY, AWC, PK, AB: writing (review & editing)’. In addition, the following was added to the Acknowledgements: ‘We would like to thank Michael D. Howell for his role in reviewing our manuscript and Viknesh Sounderajah for his input in the conception of this review’.

This has been corrected in both the PDF and HTML versions of the Article.

