# Supplementary material for: Concordance of randomised controlled trials for artificial intelligence interventions with the CONSORT-AI reporting guidelines
Source: Nat Commun. 2024 Feb 22;15:1619. doi: 10.1038/s41467-024-45355-3 (PMC10883966; doi:10.1038/s41467-024-45355-3)
Supplement: Supplementary file 1 — Supplementary Information [file 41467_2024_45355_MOESM1_ESM.pdf]

PRISMA 2020 Checklist

Section and Topic	Item #	Checklist item	Location where item is reported
**TITLE**			
Title	1	Identify the report as a systematic review.	Pg 3
**ABSTRACT**			
Abstract	2	See the PRISMA 2020 for Abstracts checklist.	Pg 3
**INTRODUCTION**			
Rationale	3	Describe the rationale for the review in the context of existing knowledge.	Pg 4
Objectives	4	Provide an explicit statement of the objective(s) or question(s) the review addresses.	Pg 4
**METHODS**			
Eligibility criteria	5	Specify the inclusion and exclusion criteria for the review and how studies were grouped for the syntheses.	Pg 5
Information sources	6	Specify all databases, registers, websites, organisations, reference lists and other sources searched or consulted to identify studies. Specify the date when each source was last searched or consulted.	Pg 5
Search strategy	7	Present the full search strategies for all databases, registers and websites, including any filters and limits used.	Pg 5, Appendix 2
Selection process	8	Specify the methods used to decide whether a study met the inclusion criteria of the review, including how many reviewers screened each record and each report retrieved, whether they worked independently, and if applicable, details of automation tools used in the process.	Pg 5-6
Data collection process	9	Specify the methods used to collect data from reports, including how many reviewers collected data from each report, whether they worked independently, any processes for obtaining or confirming data from study investigators, and if applicable, details of automation tools used in the process.	Pg 5-6
Data items	10a	List and define all outcomes for which data were sought. Specify whether all results that were compatible with each outcome domain in each study were sought (e.g. for all measures, time points, analyses), and if not, the methods used to decide which results to collect.	Pg 6
	10b	List and define all other variables for which data were sought (e.g. participant and intervention characteristics, funding sources). Describe any assumptions made about any missing or unclear information.	Pg 6
Study risk of bias assessment	11	Specify the methods used to assess risk of bias in the included studies, including details of the tool(s) used, how many reviewers assessed each study and whether they worked independently, and if applicable, details of automation tools used in the process.	N/A (Pg 6)
Effect measures	12	Specify for each outcome the effect measure(s) (e.g. risk ratio, mean difference) used in the synthesis or presentation of results.	Pg 6
Synthesis methods	13a	Describe the processes used to decide which studies were eligible for each synthesis (e.g. tabulating the study intervention characteristics and comparing against the planned groups for each synthesis (item #5)).	Pg 6-7
	13b	Describe any methods required to prepare the data for presentation or synthesis, such as handling of missing summary statistics, or data conversions.	Pg 6-7
	13c	Describe any methods used to tabulate or visually display results of individual studies and syntheses.	Pg 6-7
	13d	Describe any methods used to synthesize results and provide a rationale for the choice(s). If meta-analysis was performed, describe the model(s), method(s) to identify the presence and extent of statistical heterogeneity, and software package(s) used.	Pg 6-7
	13e	Describe any methods used to explore possible causes of heterogeneity among study results (e.g. subgroup analysis, meta-regression).	N/A
	13f	Describe any sensitivity analyses conducted to assess robustness of the synthesized results.	N/A
Reporting bias assessment	14	Describe any methods used to assess risk of bias due to missing results in a synthesis (arising from reporting biases).	N/A (Pg 6, ROB briefly reported in

Section and Topic	Item #	Checklist item	Location where item is reported
			Appendix 5 as part of CONSORT)
Certainty assessment	15	Describe any methods used to assess certainty (or confidence) in the body of evidence for an outcome.	Pg 6 (use of CONSORT and CONSORT AI)
**RESULTS**			
Study selection	16a	Describe the results of the search and selection process, from the number of records identified in the search to the number of studies included in the review, ideally using a flow diagram.	Pg 8-9, Fig 1
	16b	Cite studies that might appear to meet the inclusion criteria, but which were excluded, and explain why they were excluded.	Pg 8-9
Study characteristics	17	Cite each included study and present its characteristics.	Pg 9-10, Fig 2-3 Appendix 4
Risk of bias in studies	18	Present assessments of risk of bias for each included study.	N/A
Results of individual studies	19	For all outcomes, present, for each study: (a) summary statistics for each group (where appropriate) and (b) an effect estimate and its precision (e.g. confidence/credible interval), ideally using structured tables or plots.	Pg 9-17, Table 2
Results of syntheses	20a	For each synthesis, briefly summarise the characteristics and risk of bias among contributing studies.	N/A (ROB briefly reported in Appendix 5 as part of CONSORT)
	20b	Present results of all statistical syntheses conducted. If meta-analysis was done, present for each the summary estimate and its precision (e.g. confidence/credible interval) and measures of statistical heterogeneity. If comparing groups, describe the direction of the effect.	Pg 9-17, Table 2. No meta-analysis was done.
	20c	Present results of all investigations of possible causes of heterogeneity among study results.	N/A
	20d	Present results of all sensitivity analyses conducted to assess the robustness of the synthesized results.	N/A
Reporting biases	21	Present assessments of risk of bias due to missing results (arising from reporting biases) for each synthesis assessed.	Table 3 (CONSORT-AI); Appendix 5 (CONSORT)
Certainty of evidence	22	Present assessments of certainty (or confidence) in the body of evidence for each outcome assessed.	Pg 13
**DISCUSSION**			

PRISMA 2020 Checklist

Section and Topic	Item #	Checklist item	Location where item is reported
Discussion	23a	Provide a general interpretation of the results in the context of other evidence.	Pg 18-21
	23b	Discuss any limitations of the evidence included in the review.	Pg 21-22
	23c	Discuss any limitations of the review processes used.	Pg 21-22
	23d	Discuss implications of the results for practice, policy, and future research.	Pg 22-23
**OTHER INFORMATION**			
Registration and protocol	24a	Provide registration information for the review, including register name and registration number, or state that the review was not registered.	Pg 5
	24b	Indicate where the review protocol can be accessed, or state that a protocol was not prepared.	Pg 5
	24c	Describe and explain any amendments to information provided at registration or in the protocol.	Pg 7
Support	25	Describe sources of financial or non-financial support for the review, and the role of the funders or sponsors in the review.	Pg 24
Competing interests	26	Declare any competing interests of review authors.	Pg 24
Availability of data, code and other materials	27	Report which of the following are publicly available and where they can be found: template data collection forms; data extracted from included studies; data used for all analyses; analytic code; any other materials used in the review.	Pg 24, Appendix 4

From: Page MJ, McKenzie JE, Bossuyt PM, Boutron I, Hoffmann TC, Mulrow CD, et al. The PRISMA 2020 statement: an updated guideline for reporting systematic reviews. BMJ 2021;372:n71. doi: 10.1136/bmj.n71

For more information, visit: http://www.prisma-statement.org/

MEDLINE search

Database: Ovid MEDLINE(R) ALL <1946 to September 16, 2022>
Search Strategy:
----
1 exp artificial intelligence/ (156000)
2 exp decision making, computer-assisted/ (131681)
3 decision support systems, clinical/ (9194)
4 (((machine or deep or ensemble) adj3 learning) or artificial intelligence*).ti,ab. (105081)
5 ((convolutional or neural or deep) adj1 net*).ti,ab. (81648)
6 (naive bayes or random forest or multilayer perceptron).ti,ab. (18287)
7 ((reinforcement or semi?supervised or self?supervised) adj2 learning).ti,ab. (4945)
8 Automation/ (19804)
9 Computer systems/ (13545)
10 1 or 2 or 3 or 4 or 5 or 6 or 7 or 8 or 9 (399730)
11 randomized controlled trial.pt. (577250)
12 controlled clinical trial.pt. (95029)
13 randomi#ed.ti,ab. (744523)
14 clinical trials as topic.sh. (200369)
15 exp animals/ not humans.sh. (5047902)
16 11 or 12 or 13 or 14 (1200408)
17 16 not 15 (1160986)
18 10 and 17 (11811)
19 limit 18 to (english language and yr="2020 -Current") (2227)

Embase search

Database: Embase <1974 to 2022 September 16>
Search Strategy:

1	exp artificial intelligence/ (66272)
2	exp decision support system/ (31583)
3	(((machine or deep or ensemble) adj3 learning) or artificial intelligence*).tw. (124216)
4	((convolutional or neural or deep) adj1 net*).tw. (96842)
5	(naive bayes or random forest or multilayer perceptron).tw. (22580)
6	((reinforcement or semi?supervised or self?supervised) adj2 learning).tw. (5504)
7	automation/ (64498)
8	computer system/ (26317)
9	1 or 2 or 3 or 4 or 5 or 6 or 7 or 8 (349431)
10	randomized controlled trial/ (727812)
11	controlled clinical trial/ or "controlled clinical trial (topic)"/ (479281)
12	randomi?ed.tw. (1066748)
13	10 or 11 or 12 (1462294)
14	exp animal/ not human.sh. (5142839)
15	13 not 14 (1410594)
16	9 and 15 (9414)
17	limit 16 to (english language and yr="2020 -Current") (3455)

Cochrane Central search

Search Name:
Date Run: 19/09/2022 10:52:00
Comment:

ID Search Hits
#1 MeSH descriptor: [Artificial Intelligence] explode all trees 1466
#2 MeSH descriptor: [Decision Making, Computer-Assisted] explode all trees 4469
#3 MeSH descriptor: [Decision Support Systems, Clinical] this term only 450
#4 ((((machine or deep or ensemble) near/3 learning) or artificial intelligence*)):ti,ab,kw 3445
#5 (((convolutional or neural or deep) near/1 net*)):ti,ab,kw 1598
#6 ((naive bayes or random forest or multilayer perceptron)):ti,ab,kw 725
#7 (((reinforcement or semi?supervised or self?supervised) near/2 learning)):ti,ab,kw 198
#8 MeSH descriptor: [Automation] this term only 287
#9 MeSH descriptor: [Computer Systems] this term only 151
#10 #1 or #2 or #3 or #4 or #5 or #6 or #7 or #8 or #9 10714
#11 ("randomized-controlled trial"):pt 556044
#12 ("controlled clinical trial"):pt 91815
#13 (randomi?ed):ti,ab,kw 1021292
#14 #11 or #12 or #13 1280147
#15 #10 and #14 with Publication Year from 2020 to present, in Trials 2532

PRISMA-S Checklist

Section/topic	#	Checklist item	Location(s) Reported
**INFORMATION SOURCES AND METHODS**			
Database name	1	Name each individual database searched, stating the platform for each.	Pg 5
Multi-database searching	2	If databases were searched simultaneously on a single platform, state the name of the platform, listing all of the databases searched.	Pg 75
Study registries	3	List any study registries searched.	Pg 5, Appendix 2
Online resources and browsing	4	Describe any online or print source purposefully searched or browsed (e.g., tables of contents, print conference proceedings, web sites), and how this was done.	Pg 5 (clinical trial registry platform and ClinicalTrials.gov)
Citation searching	5	Indicate whether cited references or citing references were examined, and describe any methods used for locating cited/citing references (e.g., browsing reference lists, using a citation index, setting up email alerts for references citing included studies).	Pg 5 (reference list screening)
Contacts	6	Indicate whether additional studies or data were sought by contacting authors, experts, manufacturers, or others.	Not performed
Other methods	7	Describe any additional information sources or search methods used.	Pg 5 (manual searching)
**SEARCH STRATEGIES**			
Full search strategies	8	Include the search strategies for each database and information source, copied and pasted exactly as run.	Appendix 2
Limits and restrictions	9	Specify that no limits were used, or describe any limits or restrictions applied to a search (e.g., date or time period, language, study design) and provide justification for their use.	Pg 5
Search filters	10	Indicate whether published search filters were used (as originally designed or modified), and if so, cite the filter(s) used.	Pg 5 (Cochrane RCT

			sensitivity and precision maximizing filter)
Prior work	11	Indicate when search strategies from other literature reviews were adapted or reused for a substantive part or all of the search, citing the previous review(s).	Not used (p5)
Updates	12	Report the methods used to update the search(es) (e.g., rerunning searches, email alerts).	Not performed (p5)
Dates of searches	13	For each search strategy, provide the date when the last search occurred.	Appendix 2
**PEER REVIEW**			
Peer review	14	Describe any search peer review process.	Not performed
**MANAGING RECORDS**			
Total Records	15	Document the total number of records identified from each database and other information sources.	Figure 1, Appendix
Deduplication	16	Describe the processes and any software used to deduplicate records from multiple database searches and other information sources.	Pg 5 (Covidence systematic review software)

PRISMA-S: An Extension to the PRISMA Statement for Reporting Literature Searches in Systematic Reviews
Rethlefsen ML, Kirtley S, Waffenschmidt S, Ayala AP, Moher D, Page MJ, Koffel JB, PRISMA-S Group.
Last updated February 27, 2020.
