# Supplementary material for: Concordance of randomised controlled trials for artificial intelligence interventions with the CONSORT-AI reporting guidelines
Source: Nat Commun. 2024 Feb 22;15:1619. doi: 10.1038/s41467-024-45355-3 (PMC10883966; doi:10.1038/s41467-024-45355-3)
Supplement: Supplementary file 3 — Description of Additional Supplementary Files [file 41467_2024_45355_MOESM3_ESM.pdf]

Description of Additional Supplementary Files

Title: Supplementary Data 1

Description: Study characteristics of included randomised controlled trials.

Title: Supplementary Data 2

Description: Concordance of studies with individual CONSORT-AI items.
